# The correct nomenclature of Zirelí sign in the propaedeutics of pityriasis versicolor (*in memoriam*)^☆^^☆☆^

**DOI:** 10.1016/j.abd.2021.04.004

**Published:** 2021-07-19

**Authors:** John Verrinder Veasey, Priscila Marques de Macedo, José Roberto Amorim, Rosane Orofino-Costa

**Affiliations:** a Santa Casa de Misericórdia de São Paulo, São Paulo, SP, Brazil; b Instituto Nacional de Infectologia Evandro Chagas, Fiocruz, Rio de Janeiro, RJ, Brazil; c Universidade Federal de Alagoas, Maceió, AL, Brazil; d Hospital Universitário Pedro Ernesto, Universidade do Estado do Rio de Janeiro, Rio de Janeiro, RJ, Brazil

**Keywords:** Diagnosis, Malassezia, Mycoses, Tinea versicolor

## Abstract

Aiming at disclosing the semiotic method used in the diagnosis of pityriasis versicolor, the authors go through the history of the creation of Zirelí sign, describing the method, its usefulness and practicality in dermatological clinical practice, whether public or private, and to give credit to the author of this semiological maneuver, *in memoriam*.

Pityriasis versicolor (PV) is a superficial mycosis, usually chronic and recurrent, caused by yeast species of the genus Malassezia, which are part of the human skin microbiota. Dermatologists do not always have access to a specific laboratory test (direct mycological test) for diagnosis, which is attained, most of the time, by the highly suggestive clinical presentation, characterized by hypochromic, hyperchromic, or erythematous macules, covered by fine, furfuraceous scaling (floury). There are two semiotic maneuvers that disclose this fine scaling that is so characteristic of PV: the scratch sign, also known as Besnier sign; and the lateral stretching sign, called Zirelí sign. The latter is the better known among Brazilian dermatologists.^1^, ^2^

PV affects individuals worldwide; however, it is more common in countries with tropical and subtropical climates, such as Brazil, where its incidence can reach up to 40% to 50% of the population in some regions.^3^ Therefore, it was necessary to develop a safe, reliable, and accessible semiological maneuver in both private clinics and public services, based on clinical manifestations, especially in cases where other differential diagnoses must be considered, such as pityriasis alba, pityriasis rosea, syphilis, and indeterminate leprosy, among others.

Zirelí de Oliveira Valença, a Brazilian dermatologist, was born in the municipality of São José da Laje, state of Alagoas, on January 5, 1934, and died at the age of 86, on December 23, 2020. He graduated from the School of Medicine of the Universidade Federal de Alagoas in 1960 (Fig. 1). In 1961, he did a two-month internship at A. C. Camargo Hospital in São Paulo and then at Hospital das Clínicas, Universidade de São Paulo, when he met Professor Sebastião de Almeida Sampaio, and the friendship between the two physicians was initiated. In 1962 he became a professor at the School of Medicine in Alagoas, having retired at the age of 70. In 1963 he became the head of the outpatient clinic of Skin Diseases, and later also of the infirmary, at Santa Casa de Misericórdia in Maceió, where he continued to work until he was 80 years old. In the municipality of São José da Laje, where he was born, he was awarded a tribute from the local Mayor, who gave his name to the Basic Health Unit for the service he provided to the most disadvantaged.

**Figure 1:**
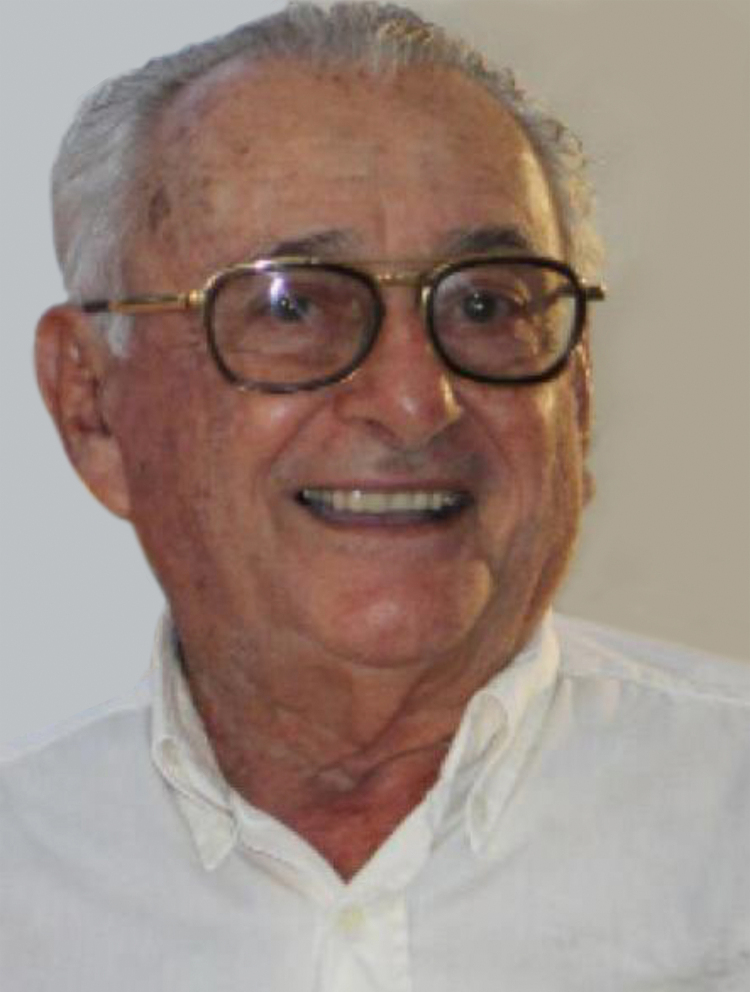
Dr. Zirelí de Oliveira Valença, Brazilian dermatologist who described the Zirelí sign for the clinical diagnosis of pityriasis versicolor.

Professor Zirelí, who was a born observer and researcher, personally introduced to Professor Sampaio, at the beginning of the 1970s, during a Dermatology Meeting held in Maceió, Alagoas, the result of a research carried out by him in more than 1,000 patients concerning a maneuver that consisted in laterally stretching the skin, which was easy to perform and helped in the clinical diagnosis of PV. Both decided to present it during a scientific meeting of the specialty, when the eponym was “christened” by Prof. Sampaio as Zirelí sign, in honor of its creator. This sign was officially presented for the first time in 1974, at the III World Congress of Tropical Dermatology held in the city of São Paulo, as a characteristic semiotic sign that consists of demonstrating the furfuraceous aspect of the scaling, present in the lesions, by stretching the skin. around the suspected PV lesion (Fig. 2). Later, in 1979, during the XXXV Brazilian Congress of Dermatology and I Brazilian Meeting of Sanitary Dermatology, held in the municipality of Poços de Caldas, state of Minas Gerais, the free topic “New maneuver for the diagnosis of pityriasis versicolor” was presented by Prof. Zirelí.

**Figure 2:**
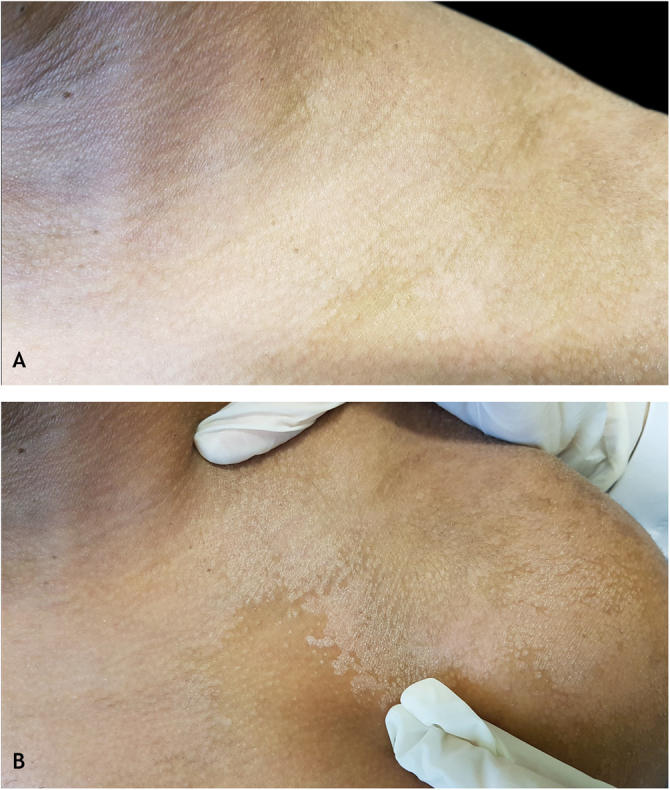
Zirelí propaedeutic maneuver in a pityriasis versicolor lesion on the left shoulder: (A) at rest; (B) after stretching (Zirelí sign), causing furfuraceous scales to detach and making the lesions evident.

The *ZiReLí* sign was first published in the book *Dermatologia Básica*, by Sampaio, in its 2^nd^ edition in 1978, but with the wrong spelling, “ZiLeRi” (pronounced ZilÉri).^4^ Probably from then on, the eponym was written incorrectly for years in several articles and chapters of national and foreign books.^2^, ^5^, ^6^, ^7^, ^8^, ^9^, ^10^, ^11^ A publication by the Ministry of Health in 2002 also contains the incorrect spelling, even though Professor Sampaio, a friend of Professor Zirelí, is one of the authors.^6^

For many years, the origin of the author of this maneuver has been discussed in academic circles of Brazilian dermatology, with most believing it was a “surname” of French origin due to the fact that the last syllable was the tonic one, except for those who already knew him, and who started to spell the eponym correctly.^1^, ^3^, ^7^, ^12^, ^13^, ^14^, ^15^, ^16^, ^17^, ^18^, ^19^

It is noteworthy that within the same article, the Portuguese version has the correct spelling as ‘Zirelí sign’, but in the English language version, it is written Zileri, which very well characterizes the misnomer in the academic environment.^8^ Sometimes the same authors who use the correct spelling in one article change the letters in others.^5^, ^9^, ^13^, ^14^^,^^15^

Santana et al., in 2013, showed the precision this semiological sign has in the clinical diagnosis of PV by presenting the statistical evidence of the correlation between the Zirelí sign and the positive direct mycological test for PV using Porto’s method (using transparent adhesive tape) (p < 0.05). However, they incorrectly spelled the name of the author of the reported maneuver.^8^, ^20^

Foreign books and articles, mostly written in English and French, rarely mention this semiotic maneuver in the clinical description of PV, perhaps because it is less frequent in these areas of the globe (about 0.8% –1.1%) or even due to lack of knowledge about it.^1^, ^21^, ^22^ Some mention the scratch sign, also known as the Besnier sign, which is no longer used today due to the exposure of the examiner's nail to the parasitized scales, as well as the possibility of contamination of the patient's injured skin (breakage of the skin barrier) by the examiner's nail.^3^, ^10^, ^17^, ^18^, ^19^

In this article, the authors intend to reinforce the correct spelling of the Zirelí sign and disseminate it beyond Brazilian borders, as it is a cost-effective method, easy to perform in daily dermatological practice, as well as to honor this Brazilian dermatologist who, as a good scientist, left this legacy to us, creating a simple and safe maneuver, routinely practiced in dermatological outpatient clinics and offices.

## Financial support

None declared.

## Authors' contributions

John Verrinder Veasey: Participation in the design and planning; collection of data; preparation and writing of the manuscript; critical review of the literature and of the manuscript.

Priscila Marques de Macedo: Participation in the design and planning; collection of data; preparation and writing of the manuscript; critical review of the literature and of the manuscript.

José Roberto Amorim: Participation in the design and planning; collection of data; preparation and writing of the manuscript; critical review of the literature and of the manuscript.

Rosane Orofino-Costa: Participation in the design and planning; collection of data; preparation and writing of the manuscript; critical review of the literature and of the manuscript.

## Conflicts of interest

None declared.
